# NEuroBioStand project: a European metrological research framework for standardising p‐tau measurements in plasma

**DOI:** 10.1002/alz.092203

**Published:** 2025-01-09

**Authors:** Chiara Giangrande, Hélène Vaneeckhoutte, Vincent Delatour, Joëlle Vinh, Yann Verdier, Eugeen Vanmechelen, Peter Koertvelyessy, Kaj Blennow, Henrik Zetterberg, Johan Gobom

**Affiliations:** ^1^ Laboratoire national de métrologie et d'essais, Paris France; ^2^ ESPCI‐CNRS, Paris France; ^3^ ADx NeuroSciences NV, Technologiepark 94, Gent Belgium; ^4^ Charité Universitätsmedizin Berlin, Berlin Germany; ^5^ Clinical Neurochemistry Laboratory, Sahlgrenska University Hospital, Mölndal Sweden; ^6^ Department of Psychiatry and Neurochemistry, Institute of Neuroscience and Physiology, The Sahlgrenska Academy, University of Gothenburg, Mölndal, Gothenburg Sweden; ^7^ Department of Neurodegenerative Disease, UCL Queen Square Institute of Neurology, University College London, London, ‐ UK; ^8^ Wisconsin Alzheimer’s Disease Research Center, University of Wisconsin School of Medicine and Public Health, Madison, WI USA; ^9^ UK Dementia Research Institute, University College London, London UK; ^10^ Hong Kong Center for Neurodegenerative Diseases, Clear Water Bay Hong Kong; ^11^ Department of Psychiatry and Neurochemistry, Institute of Neuroscience and Physiology, The Sahlgrenska Academy at the University of Gothenburg, Mölndal Sweden; ^12^ Department of Psychiatry and Neurochemistry, Institute of Neuroscience and Physiology, The Sahlgrenska Academy, University of Gothenburg, Mölndal Sweden; ^13^ Clinical Neurochemistry Laboratory, Sahlgrenska University Hospital, Gothenburg, VGR Sweden

## Abstract

**Background:**

NEuroBioStand is an EU‐funded project aimed at developing a metrological research framework for standardising blood‐based biomarkers of neurodegenerative diseases (NDDs) with the objective of implementation and commercialisation of promising assays for NDD biomarkers fulfilling requirements of the in vitro diagnostic regulation (IVDR).

P‐tau is currently included in the AT(N) framework for AD diagnosis together with other biomarkers and amyloid‐β and tau in CSF have been developed into regulatory approved biomarkers in CSF. The standardisation of the measurements for this biomarker is important to establish common cut‐off values and reference ranges.

**Method:**

Recombinant phosphorylated protein and synthetic phosphopeptide materials were sourced as candidate primary calibrators together with their labelled internal standards. Purity was evaluated for the protein and peptide materials by high resolution mass spectrometry coupled to liquid chromatography (LC‐HRMS) and mass fraction was determined by amino acid analysis (AAA). A candidate reference measurement procedure (RMP) is being developed by LC‐HRMS for high accuracy measurement of different phosphorylated epitopes in plasma.

**Result:**

The first step towards the standardisation of this biomarker is the definition of the measurand: NEuroBioStand consortium has defined the clinically relevant phosphorylated isoforms to develop protein and peptide candidate primary calibrators. P‐tau181, P‐tau217 and P‐tau231 have been prioritized and fit for purpose calibrators have been characterised in terms of purity and mass fraction by amino acids analysis and LC‐MS. In particular, the full characterisation of the phosphorylation status of protein candidate primary calibrators has been carried out to determine the site occupancy of the most relevant phosphorylation sites by using the SI‐traceable quantified phosphorylated peptide materials. The development of a multiplexing SI‐traceable candidate RMP in plasma is in progress with the aim of standardising concentrations of the prioritised phosphorylated epitopes and thus calculating the ratio of phosphorylated to non‐phosphorylated fragments.

**Conclusion:**

NEuroBioStand consortium gathers national metrology institutes, clinicians, academics and IVD‐providers to achieve traceability to SI units for fluid biomarkers in NDD. The development of a RMP will allow the certification of candidate matrix‐based reference materials that will be used to harmonise measurements of P‐tau in plasma across different analytical platforms, thus standardising cut‐off values and reference ranges.

